# Optimal authoritative risk assessment score of Cancer-associated venous thromboembolism for hospitalized medical patients with lung Cancer

**DOI:** 10.1186/s12959-021-00339-x

**Published:** 2021-12-04

**Authors:** Wei Xiong, Yunfeng Zhao, He Du, Yanmin Wang, Mei Xu, Xuejun Guo

**Affiliations:** 1 Department of Pulmonary and Critical Care Medicine, Xinhua Hospital, Shanghai Jiaotong University School of Medicine,Shanghai, No. 1665, Kongjiang Road, Yangpu District, Shanghai, 200092 China; 2 Department of Pulmonary and Critical Care Medicine, Punan Hospital, Pudong New District, Shanghai, China; 3 Department of Medical Oncology, Shanghai Pulmonary Hospital, Tongji University School of Medicine, Shanghai, China; 4 Department of General Medicine, North Bund Community Health Service Center, Hongkou District, Shanghai, China

**Keywords:** Lung cancer, Venous thromboembolism, Cancer-associated VTE, Risk assessment score, Hospitalized medical patients

## Abstract

**Background:**

Cancer-associated venous thromboembolism (VTE) is common in patients with primary lung cancer. It has been understudied which authoritative risk assessment score of cancer-associated VTE is optimal for the assessment of VTE development in hospitalized medical patients with lung cancer.

**Methods:**

Patients with lung cancer who had undergone computed tomography pulmonary angiography (CTPA), compression ultrasonography (CUS) of lower and upper extremities, and/or planar ventilation/perfusion (V/Q) scan to confirm the presence or absence of VTE during a medical hospitalization were retrospectively reviewed. Based on the actual prevalence of VTE among all patients, the possibility of VTE were reassessed with the Khorana score, the PROTECHT score, the CONKO score, the ONKOTEV score, the COMPASS-CAT score, and the CATS/MICA score, to compare their assessment accuracy for VTE development.

**Results:**

A total of 1263 patients with lung cancer were incorporated into the final analysis. With respect to assessment efficiency for VTE occurrence, the scores with adjusted agreement from highest to lowest were the ONKOTEV score (78.6%), the PROTECHT score (73.4%), the CONKO score (72.1%), the COMPASS-CAT score (71.7%), the Khorana score (70.9%), and the CATS/MICA score (60.3%). The ONKOTEV score had the highest Youden index which was 0.68, followed by the PROTECHT score (0.58), the COMPASS-CAT score (0.56), the CONKO score (0.55), the Khorana score (0.53), and the CATS/MICA score (0.23).

**Conclusions:**

Among the Khorana score, the PROTECHT score, the CONKO score, the ONKOTEV score, the COMPASS-CAT score, and the CATS/MICA score which are approved by authoritative guidelines, the ONKOTEV score is optimal for the assessment of VTE development in hospitalized medical patients with lung cancer.

## Introduction

Venous thromboembolism (VTE) is broadly defined as pulmonary embolism (PE), deep venous thrombosis (DVT), superficial vein thrombosis (SVT), and/or splanchnic vein thrombosis (SPVT), whereas narrowly defined as PE and/or DVT. Cancer-associated VTE is a common complication that threatens the life of adult patients with cancer. Patients with cancer are four to seven times more likely to develop cancer-associated VTE than patients without cancer. Established cancer-associated VTE is an important cause of morbidity and the second leading cause of mortality for patients with cancer [1–5]. Although its incidence and mortality have shown a declining trend, primary lung cancer still remains the second most common cancer type with the highest mortality rate globally [6, 7]. Rates of VTE prevalence ranged from 7 to 13% among patients with lung cancer [8]. The occurrence of VTE events was an indicator that was significantly associated with an increased risk of mortality in patients with lung cancer [9, 10]. Under such circumstances, missing a diagnosis of VTE or the need of thromboprophylaxis could be devastating, whereas frequent VTE diagnostic tests and/or thromboprophylaxis for all lung cancer patients would significantly increase unnecessary burden. Accordingly, the risk assessment of VTE is imperative prior to the diagnostic tests and/or thromboprophylaxis of cancer-associated VTE for patients with lung cancer.

A time-dependent association between VTE and cancer has been observed after cancer diagnosis [2]. Risk of VTE should be assessed initially and periodically thereafter for patients with cancer, particularly at the initiation of systemic anticancer therapy or during hospitalization [3]. The increased risk of VTE can be affected by a variety of risk factors such as cancer site, metastasis stage, surgery, hospitalization, central venous catheters, systemic anticancer therapy, history of previous VTE, obesity, immobility, platelet, leucocyte, and D-dimer [2]. However, single risk factor does not reliably identify patients with cancer at high risk of VTE development. In cancer patients treated with systemic therapy, the assessment of VTE development and thromboprophylaxis need are usually performed with validated risk assessment scores [2, 3].

The contemporary VTE risk assessment scores for ambulatory patients with cancer in the authoritative guidelines [2, 3] mainly comprise the Khorana score [11], the Vienna score [12], the PROTECHT score [13], the CONKO score [14], the ONKOTEV score, [15] the COMPASS-CAT score [16], the Tic-Onco score [17], and the CATS/MICA score [18]. The Vienna score, the PROTECHT score, the CONKO score, and the ONKOTEV score are modified Khorana risk score (KRS). A few studies compared the performance of different VTE risk assessment scores in patients with cancer. In a study comparing the Khorana, Vienna, PROTECHT, and CONKO scores in a prospective cohort of 876 patients with advanced cancer, the results showed that the patients with high risk score had a significantly increased risk of VTE by using the Vienna or PROTECHT scores [19].

With respect to lung cancer, another study comparing the predictive efficiency among the Khorana, PROTECHT, CONKO and COMPASS-CAT scores in 118 lung cancer patients showed that only the COMPASS-CAT score identified 100% of patients who developed VTE, being the most accurate risk assessment model of VTE occurrence in patients with lung cancer [20]. Nevertheless, since routine VTE screening was not performed in the study, the asymptomatic VTE may have been missed, interfering with the confidence of the conclusion. Besides, the sample size(*n* = 118) was too poor to draw a convincing conclusion.

Taken together, despite the aforementioned risk scores of VTE have been validated in ambulatory patients with cancer or lung cancer, their roles in hospitalized patients with cancer have been understudied. Besides, which of them is the most appropriate one to assess the VTE development for hospitalized patients with lung cancer remains unknown. Accordingly, the current study was performed to compare the assessment accuracy of VTE development by the risk assessment scores approved by the authoritative guidelines in hospitalized medical patients with lung cancer [2, 3].

## Methods

### Study design

A retrospective study was performed to explore which one of the currently authoritative risk assessment scores for VTE including PE and/or DVT had the optimal assessment accuracy for the development of VTE in hospitalized medical patients with primary lung cancer. We reviewed consecutive patients with lung cancer who had undergone VTE investigation including computed tomography pulmonary angiography (CTPA), compression ultrasonography (CUS) of lower and upper extremities, and/or planar ventilation/perfusion (V/Q) scan [21, 22] during a medical hospitalization which implied a higher probability of VTE than ambulatory outpatients. Medical hospitalization denotes the hospitalization in which chemotherapy, radiotherapy, targeted therapy, immunotherapy, and/or other medical treatment were administered to patients. Patients underwent all of the aforementioned three investigations unless there was a contraindication to CTPA. The patients were assigned into the lung cancer (LC) and lung cancer-VTE (LC-VTE) groups according to whether or not they had been diagnosed with VTE till the present study or death prior to the present study after the diagnosis of lung cancer. In the meantime of VTE diagnostic investigation, all patients received thromboprophylaxis through discharge unless there was a contraindication. The follow-up period commenced from the diagnosis of lung cancer to the present study or the death of patients, whereas the post hoc analysis period initiated from lung cancer diagnosis to the last time of VTE testing in hospitalization prior to the present study.

In the current study, the likelihood of VTE in patients were reassessed with the Khorana score, the PROTECHT score, the CONKO score, the ONKOTEV score, the COMPASS-CAT score, and the CATS/MICA score, thereby comparing their assessment accuracy for the development of VTE. The Khorana score comprises variables including primary site of cancer (very high risk [2 points] or high risk [1 point]), prechemotherapy platelet count of 350 × 10^9^ /L or more (1 point), hemoglobin level less than 100 g/L and/or use of red cell growth factors (1 point), leukocyte count more than 11 × 10^9^ /L (1 point), and body mass index (BMI) of 35 kg/m^2^ or more (1 point). A total score of 3 or more was defined as high risk of VTE [11]. The PROTECHT score consists of Khorana score, gemcitabine chemotherapy (1point), and platinum-based chemotherapy (1point). A total score of 3 or more was defined as high risk of VTE [13]. The CONKO score is also a revised Khorana score in which BMI is replaced by the Eastern Cooperative Oncology Group (ECOG)/World Health Organization (WHO) performance status ≥2 (1 point). A total score of 3 or more was defined as high risk of VTE [14]. The ONKOTEV score is based on a Khorana score > 2(1 point), metastatic disease (1point), previous VTE (1point), and vascular/lymphatic macroscopic compression (1point). A total score of 2 or more was defined as high risk of VTE [15]. The COMPASS-CAT score includes anti-hormonal or anthracycline therapy (6 points), time since cancer diagnosis ≤6 months (4 points), central venous catheter (3 points), advanced stage of cancer (2 points), cardiovascular risk factors (5 points), recent hospitalization (5 points), personal history of VTE (1 point), and platelet count ≥350 × 10^9^ /L (2 points). A total score of 7 or more was defined as high risk of VTE [16]. The CATS/MICA score comprises one tumor-site category and D-dimer level. A 6-month cumulative risk of VTE ≥ 10%(or a total score ≥ 110) was defined as high risk of VTE [18].

The patients with high risk of VTE assessed by each score were defined as VTE likely, whereas those with non-high risk of VTE were defined as VTE unlikely. Then such dichotomy was contrasted with the actual presence and absence of VTE confirmed by CTPA, V/Q scan, and CUS, so as to compare the assessment accuracy for VTE development among these risk assessment scores. The parameter values at admission were adopted for the variables involved in these scores. For patients who had been diagnosed with VTE prior to the present study, the data of the hospitalization in which VTE was initially diagnosed was incorporated into the present study as one case, whereas for those who had not been diagnosed with VTE until the present study, the data in the hospitalization in which the last time of VTE diagnostic testing was performed prior to the present study was adopted as one case. For each patient, all these VTE risk assessment scores were performed post hoc based on the data in the same hospitalization. One patient could not be counted as more than one case. All data were retrieved from the Electronic Medical Record (EMR) of three hospitals in Shanghai, including Shanghai Xinhua Hospital, Shanghai Pulmonary Hospital, and Shanghai Punan Hospital. The protocol was approved by the institutional review boards of these hospitals.

### Study population

In terms of inclusion and exclusion criteria, we incorporated eligible patients into the current study. The inclusion criteria comprised: 1) all eligible patients were 18 years old or older; 2) all eligible patients had a definite histopathological diagnosis of primary lung cancer; 3) all eligible patients with lung cancer underwent CTPA, CUS and/or V/Q scan that could confirm the presence or absence of VTE during the hospitalizations of diagnoses or medical treatment of lung cancer, or an acute medical illness; 4) all eligible patients had complete information required for the study. The exclusion criteria comprised: 1) patients who had other known primary cancers apart from lung cancer were excluded; 2) patients who had a history of chronic VTE or thrombophilia were excluded; 3) patients who had undergone major surgery or trauma within previous month prior to the diagnoses of VTE were excluded.

### Statistical analyses

Comparison of measurement data between groups was performed by using T-test. The comparison of rates was performed by Chi-square test. The number of true positive (TP), false positive (FP), false negative (FN), and true negative (TN) resulted from each risk score were compared between every two risk scores. The sensitivity, specificity, positive predictive value (PPV), negative predictive value (NPV), false positive rate (FPR), false negative rate (FNR), positive likelihood ratio (PLR), negative likelihood ratio (NLR), diagnostic odds ratio (DOR), crude agreement (CA), adjusted agreement (AA), and Youden index (YI) for the assessment of VTE development were compared among the Khorana score, the PROTECHT score, the CONKO score, the ONKOTEV score, the COMPASS-CAT score, and the CATS/MICA score. SPSS 26 was used for the statistical analysis. A *P*-value being less than 0.05 was defined as statistical significance.

## Results

### Demographics and characteristics of patients

A total of 1370 patients with lung cancer from Jan, 2013 through Dec, 2020 were incorporated into the current study based on the inclusion criteria. According to the exclusion criteria, 29 patients who had other known primary cancers apart from lung cancer, 37 patients who had a history of chronic VTE or thrombophilia and 41 patients who had undergone major surgery or trauma within previous month prior to the diagnoses of VTE were excluded. Finally, 1263 patients were determined to be in the analysis of current study. The mean age of all patients was 70.4 years old. The number of female and male patients were 550 and 713, respectively. Among a total of 1263 patients with primary lung cancer who underwent VTE-confirming investigations, all patients underwent CUS and V/Q scan, and 1092 patients underwent CTPA whereas 171 patients did not due to contraindications.

Taken together, among 1263 patients with lung cancer, 173 patients (13.7%) had VTE, whereas 1090 ones had not. Among 173 patients with established VTE, 79 and 57 ones solely had PE and DVT, respectively, whereas 37 ones had both PE and DVT. For 173 patients with established PE, 155 patients were diagnosed with CTPA and/or V/Q scan, whereas 18 patients were diagnosed with sole V/Q scan due to the contraindications to CTPA. For 1090 patients whose VTE diagnoses were excluded, 937 patients had negative results of CTPA, V/Q scan and CUS, whereas 153 patients had negative results of V/Q scan and CUS due to the contraindications to CTPA. The median time from lung cancer diagnosis to the hospitalization in which the last time of VTE diagnostic testing was performed prior to the present study in LC and LC-VTE groups were 13.3(7.6–19.0) and 15.9(9.1–22.7) months, respectively.(*p* = 0.868) The median time from lung cancer diagnosis to the hospitalization in which data were analyzed in LC and LC-VTE groups were 13.3(7.6–19.0) and 11.8(5.2–18.4) months, respectively (*p* = 0.357) The demographic and clinical characteristic of patients were summarized in Table 1.

**Table 1:** Demographics and Characteristics of Patients

Variables	LC(*n* = 1090)	LC-VTE (*n* = 173)	*P* value
Age-years	68.5 (47.6–86.3)	72.2 (51.5–88.6)	0.723
Sex (female/male)-%	43.0/57.0	46.8/53.2	0.915
BMI-kg/m^2^	21.9 (17.5–27.3)	29.6 (24.3–34.7)	0.013
Smoking (Y/N)-%	40.1/59.9	50.9/49.1	0.009
Smoking index-pack/year	33.6 (21.3–45.9)	47.3 (30.6–63.7)	0.001
Histopathology-no.(%)			
Adenocarcinoma	673 (61.7)	109 (63.0)	0.537
Squamous	269 (24.7)	40 (23.1)	0.941
SCLC	123 (11.3)	17 (9.80)	0.713
Others	25 (2.30)	7 (4.10)	0.025
Stage-no.(%)			
Stage I	199 (18.3)	3 (1.70)	< 0.001
Stage II	227 (20.8)	33 (19.1)	0.985
Stage III	257 (23.6)	44 (25.4)	0.836
Stage IV	407 (37.3)	93 (53.8)	0.001
High or intermediate			
C-PTP(Y/N)-%	45.2/54.8	74.6/25.4	< 0.001
D-dimer-mg/L	3713 (2336–5369)	1331 (854–1776)	< 0.001
Platelet- × 10^9^/L	387 (226–543)	252 (157–357)	0.001
Hemoglobin-g/L	97 (83–114)	109 (91–127)	0.573
WBC- × 10^9^/L	13.1 (7.62–18.6)	7.73 (4.38–11.1)	0.001
Chemotherapy(Y/N)-%	65.8/34.2	94.2/5.8	< 0.001
PS score-point	1.35 (0.66–2.25)	2.31 (1.23–3.47)	0.005
Metastasis(Y/N)-%	37.3/62.7	53.8/46.2	0.001
Previous VTE(Y/N)-%	9.9/90.1	17.9/82.1	0.001
Vascular/lymphatic compression(Y/N)-%	70.1/29.9	90.8/9.2	< 0.001
Anti-hormonal or anthracycline therapy(Y/N)-%	2.0/98.0	1.7/98.3	0.896
Time since cancer diagnosis≤6 months(Y/N)-%	49.8/50.2	52.6/47.4	0.916
CVC(Y/N)-%	3.9/96.1	9.8/90.2	< 0.001
Cardiovascular risk factors(Y/N)-%	15.0/85.0	29.5/70.5	< 0.001
Recent hospitalization (Y/N)-%	66.5/33.5	97.7/2.3	< 0.001

Note: LC: Lung Cancer, LC-VTE:Lung Cancer and Venous Thromboembolism, no.:number, BMI: Body Mass Index, kg/m^2^: kilogram/meter^2^, Y/N:Yes/No, SCLC: Small Cell Lung Cancer, C-PTP: Clinical Pretest Probability, mg/L:milligram/liter, L:liter, WBC: White Blood Cell, PS: Performance Status, VTE:Venous Thromboembolism, CVC: Central Venous Catheter

True Positive, False Positive, False Negative, and True Negative of all Risk Scores.

**Fig. 1:**
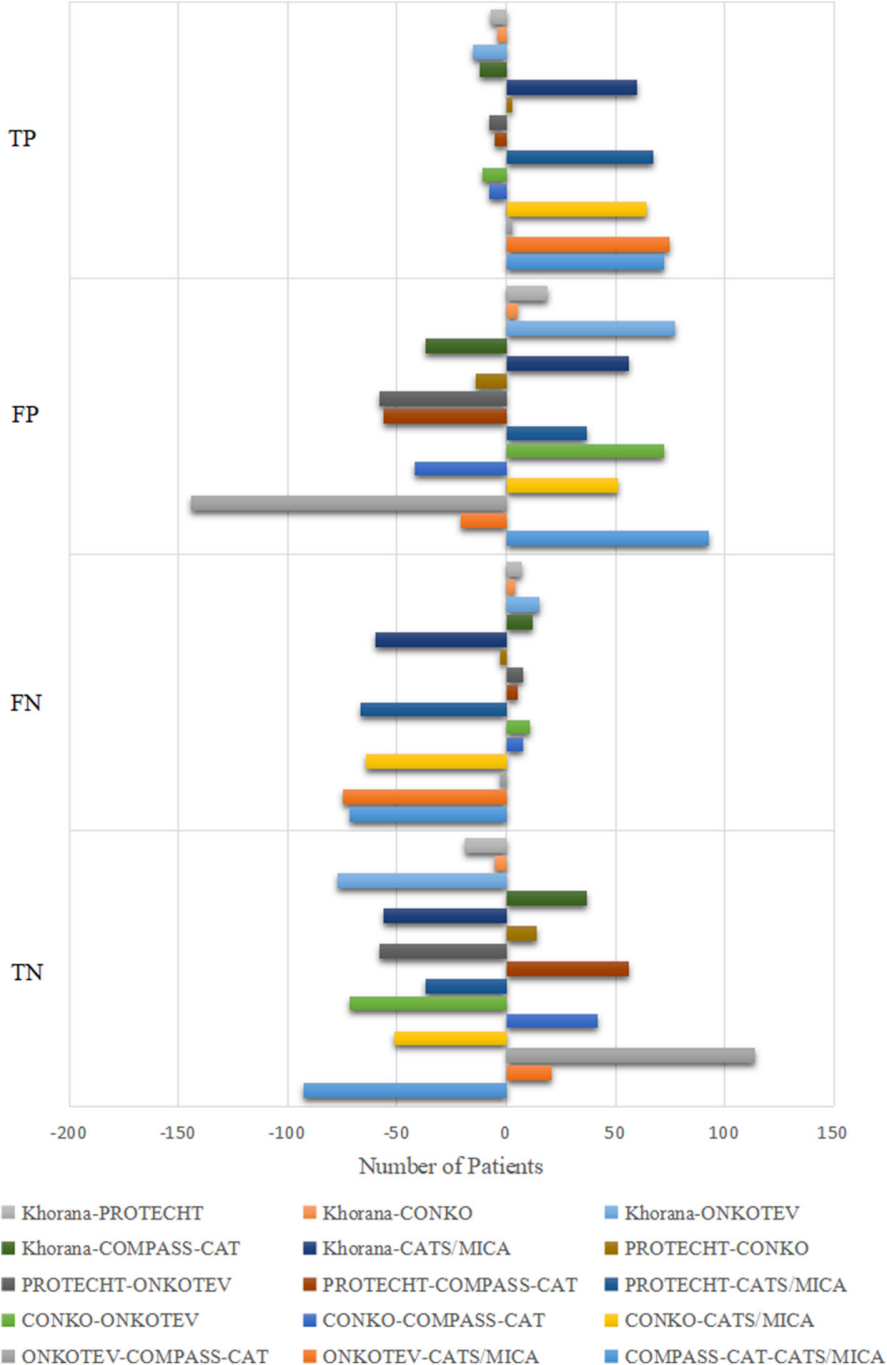
Pairwise Difference of Number of TP, FP, FN, TN Between Every Two Risk Scores. Pairwise difference of number of TP, FP, FN, TN between every two risk scores are demonstrated in Fig. 1. For example, the gray bar at the top of the first row denotes the difference of number of TP between Khorana and PROTECHT, which is −7. The orange bar right below the gray one denotes the difference of number of TP between Khorana and CONKO, which is −4. The gray bar at the top of the second row denotes the difference of number of FP between Khorana and PROTECHT, which is 19. The rest bars can be interpreted in the same manner. Note: TP:True Positive, FP: False Positive, FN: False Negative, TN: True Negative.

The VTE possibility of all patients in the final analyses were reassessed with the Khorana score, the PROTECHT score, the CONKO score, the ONKOTEV score, the COMPASS-CAT score, and the CATS/MICA score, then were contrasted with the actual VTE establishment, to determine the number of TP, FP, FN, and TN resulted from each risk score. The number of TP, FP, FN, and TN of all risk scores are demonstrated in Table 2. In contrast with the actual VTE prevalence of 13.7%, the diagnostic VTE prevalence by the Khorana score, the PROTECHT score, the CONKO score, the ONKOTEV score, the COMPASS-CAT score, and the CATS/MICA score were 27.5, 26.5, 27.4, 22.6, 31.4, and 18.3%, respectively. The Pairwise difference of number of TP, FP, FN, TN between every two risk scores are demonstrated in Fig. 1.

**Table 2:** True Positive, False Positive, False Negative, and True Negative of all Risk Scores

Variables	Khorana	PROTECHT	CONKO	ONKOTEV	COMPASS-CAT	CATS/MICA
True Positive-no.	126	133	130	141	138	66
False Positive,-no.	221	202	216	144	258	165
False Negative-no.	47	40	43	32	35	107
True Negative-no.	869	888	874	946	832	925
Diagnostic Prevalence-%	27.5	26.5	27.4	22.6	31.4	18.3
Actual Prevalence-%	13.7	13.7	13.7	13.7	13.7	13.7

Note: TP: True Positive, FP: False Positive, FN: False Negative, TN: True Negative, DP: Diagnostic Prevalence, AP: Actual Prevalence

### Comparison of assessment accuracy for VTE development among all risk scores

Base on the number of TP, FP, FN, TN of each risk score, the assessment accuracy for VTE development among all risk scores were compared. The ratio of actual VTE prevalence in patients predicted as VTE-positive over that in those predicted as VTE-negative by the Khorana score, the PROTECHT score, the CONKO score, the ONKOTEV score, the COMPASS-CAT score, and the CATS/MICA score were 7.08(36.3% vs 5.13%), 9.21(39.7% vs 4.31%), 8.02(37.6% vs 4.69%), 15.1(49.5% vs 3.27%), 8.61(34.8% vs 4.04%), 2.75(28.6% vs 10.4%), respectively. The ratio of actual VTE exclusion in patients predicted as VTE-negative over that in those predicted as VTE-positive by the Khorana score, the PROTECHT score, the CONKO score, the ONKOTEV score, the COMPASS-CAT score, and the CATS/MICA score were 1.49(94.9% vs 63.7%), 1.59(95.7% vs 60.3%), 1.53(95.3% vs 62.4%), 1.91(96.7% vs 50.5%), 1.47(96.0% vs 65.2%), 1.25(89.6% vs 71.4%), respectively.

The sensitivity, specificity, PPV, NPV, FPR, FNR, PLR, NLR, DOR, CA, AA, and Youden index for VTE assessment by all VTE risk assessment scores involved in the present study are demonstrated in Table 3. The comparison of assessment efficiency for VTE occurrence showed that the adjusted agreement of the Khorana score, the PROTECHT score, the CONKO score, the ONKOTEV score, the COMPASS-CAT score, and the CATS/MICA score were 70.9, 73.4, 72.1, 78.6, 71.7, and 60.3%, respectively. The ONKOTEV score had the highest Youden index which was 0.68, followed by the PROTECHT score (0.58), the COMPASS-CAT score (0.56), the CONKO score (0.55), the Khorana score (0.53), and the CATS/MICA score (0.23).

**Table 3:** Comparison of Assessment Accuracy for VTE Development Among all Scores

Variables	Khorana	PROTECHT	CONKO	ONKOTEV	COMPASS-CAT	CATS/MICA
Sensitivity -%	72.8	76.9	75.1	81.5	79.8	38.1
Specificity -%	79.7	81.5	80.2	86.8	76.3	84.9
PPV -%	36.3	39.7	37.6	49.5	53.5	28.6
NPV -%	94.9	95.7	85.9	96.7	96.0	89.6
FPR -%	20.3	18.5	19.8	13.2	23.7	15.1
FNR -%	27.2	23.1	24.9	18.5	20.2	61.8
PLR	3.59	4.16	3.79	6.17	3.37	2.52
NLR	0.34	0.28	0.31	0.21	0.27	0.73
DOR	10.5	14.7	12.2	29.0	12.7	3.5
CA -%	78.8	80.1	79.5	86.1	76.8	78.5
AA -%	70.9	73.4	72.1	78.6	71.7	60.3
YI	0.53	0.58	0.55	0.68	0.56	0.23

Note: PPV: Positive Predictive Value, NPV: Negative Predictive Value, FPR: False Positive Rate, FNR: False Negative Rate, PLR: Positive Likelihood Ratio, NLR: Negative Likelihood Ratio, DOR: Diagnostic Odds Ratio, CA: Crude Agreement, AA: Adjusted Agreement, YI: Youden Index

## Discussion

The results of current study revealed that the ONKOTEV score performed best in the assessment of VTE development in hospitalized medical patients with primary lung cancer, followed by the PROTECHT score, the COMPASS-CAT score, the CONKO score, the Khorana score, and the CATS/MICA score. Comparable studies similar to the current study are scarce except for the one of Rupa-Matysek et al., in which the COMPASS-CAT was most effective at predicting VTE in ambulatory outpatients with lung cancer, among the Khorana, PROTECHT, CONKO and COMPASS-CAT scores [20]. Nevertheless, the performance of COMPASS-CAT score for the assessment of VTE development was mediocre based on our findings. Since the study of Rupa-Matysek et al. did not include the ONKOTEV score, it is impossible to learn the results of comparison between the ONKOTEV score and the COMPASS-CAT score based on their study.

The ONKOTEV score outperformed the other VTE risk assessment scores involved in the current study. In the ONKOTEV study, at a multivariate analysis, it was found that a Khorana score > 2, the presence of metastatic disease status, vascular/lymphatic compression, or previous history of VTE accurately predicted the outcome of patients with cancer respectively, thereby establishing the ONKOTEV score with these four variables [15]. The Khorana score is the most classic, authoritative, and long-tested VTE risk assessment score, in which a total score > 2 represents a high risk of VTE [11]. Lung cancer patients with metastatic disease status are nearly 3 times likely to develop VTE, in contrast to those with non-metastatic status [23, 24]. In a study with respect to venous thrombosis in resected specimens for non-small cell lung cancer (NSCLC), 53.6% of cases with thrombosis were accompanied by tumor vascular infiltrating [25]. Another prospective study suggested monocyte tissue factor (TF) that was a source of TF-mediated thrombogenicity in NSCLC patients was significantly higher in patients with lymph node metastasis than those without lymph node metastasis [26]. In addition, for patients with advanced NSCLC, VTE development is associated with a previous history of VTE [27]. As a result, the combination of these three variables and a high Khorana score (> 2) could form an accurate risk assessment score for VTE development in patients with lung cancer.

Despite the authoritativeness of the Khorana score in the cancer-associated VTE risk assessment, a single Khorana score has poor performance in the assessment of VTE development for patients with lung cancer, especially for advanced lung cancer patients who may carried a high VTE risk [28–30]. Similarly, the Khorana score performed second worst in the current study. Due to a low VTE prevalence (2.2%) of the study of Khorana et al., in which the Khorana score was created, [11] the Khorana risk model appears to be a predictive tool for the identification of cancer patients at low risk of early VTE development, instead of one for patients at intermediate or high risk of VTE such as lung cancer patients.

Being basically consistent with the study of van Es N et al. [19], the PROTECHT score performed second best for the VTE assessment of patients with lung cancer in the current study. VTE is not uncommon in patients receiving chemotherapy especially platinum-based chemotherapy, of whom the majority of VTE events occur within 6 months after the initiation of chemotherapy [9, 31]. Nonetheless, since the variable “gemcitabine chemotherapy” in the PROTECHT score is unduly specific, whereas some NSCLC patients with increased VTE risk due to chemotherapy do not necessarily receive such regimen, let alone patients with small cell lung cancer. Accordingly, the efficiency of this risk score in the VTE assessment of lung cancer patients may be undermined in some degree.

The CONKO score performed moderately in Rupa-Matysek’s^20^, Alexander’s^30^, as well as the current study. The CONKO score is a modified KRS by replacing BMI with performance status (PS). Despite PS score was confirmed to be associated with an increased risk of VTE in patients with NSCLC [32], the CONKO score was almost the same as the single Khorana score. Consequently, its assessment accuracy for VTE development may be compromised similarly.

The COMPASS-CAT score performed moderately in the current study. Likewise, a large retrospective study among 3814 cancer patients including 1108 ones with lung cancer validated that the VTE assessment accuracy of COMPASS-CAT score was moderate with good negative predictive value, whereas its calibration was poor [33]. The COMPASS-CAT score is a highly comprehensive score that comprises cancer-related risk factors, patient-related predisposing risk factors, and platelets count [16]. Based on its scoring system which totally contains 28 points, many patients with lung cancer can easily reach a score of 7 that is the cutoff value of COMPASS-CAT score. Accordingly, it is a secure score which seldom misses VTE identification or thromboprophylaxis, whereas may generate excessive unnecessary VTE investigations or thromboprophylaxis. A cut-off value more than 7 points may improve its assessment accuracy of VTE development [20, 34]. Besides, the complexity of the score makes it inconvenient for clinicians to use.

The CATS/MICA score that is composed of type of cancer and D-dimer levels is a user-friendly model for the risk evaluation of cancer-associated VTE [34]. Nevertheless, being similar to its disappointing performance in the current study, the CATS/MICA score had no predictive value for VTE in the study of Alexander et al. either [30]. Since the risk for lung cancer is fixed in the CATS/MICA score, the only variable that changes is the D-dimer level. In the CATS/MICA score, the high VTE risk is defined as 6-month cumulative VTE incidence ≥10% that is approximately corresponding to a score of 110. Since patients with lung cancer (high risk tumors) definitely have a score of approximate 50 according to the CATS/MICA score, thereby at least a score of 60 for D-dimer level is required for a total score of 110. Nevertheless, a score of 60 for D-dimer level is approximately corresponding to a D-dimer level > 7 mg/L that is significantly higher than all the cutoff value of D-dimer level in other acknowledged VTE risk assessment scores [12, 21, 22, 35]. As a result, the CATS/MICA score may cause plenty of missed diagnoses due to its simplicity and excessively high cutoff value. In terms of safety, the CATS/MICA score is not a reliable VTE assessment score for patients with lung cancer.

Of note, we did not incorporate the Vienna score and the Tic-Onco score into the current study on account of soluble P-selectin and coagulation genetic variants that are respectively involved in these two scores were not routinely assayed in the hospitals participating the current study. Accordingly, we have no idea of how accurate these two risk scores are for the assessment of VTE development in lung cancer patients, in comparison with other risk scores involved in the current study. Clinical applicability and accurate identification of VTE risk assessment scores are two essential issues highlighted by Khorana [36] and Pabinger et al. [18] To date, since these two risk scores have not been validated in an external cohort, their assessment accuracy for VTE development remain unknown for patients with lung cancer. In terms of clinical applicability, the practical value of these two risk scores are limited to date by the reason that P-selectin and coagulation genetic variants are not universally tested, irrespective of their assessment accuracy for VTE development.

Although the Khorana score was highly recommended in the guidelines, [2, 3] it does not necessarily mean that it can be applied to every occasion. (e.g. lung cancer) [28–30] Fortunately, since the Khorana score was introduced, a multitude of risk assessment models for cancer-associated VTE have sprung up. Although this has led to a blissful annoyance for clinicians to make a choice, concurrently it also provides options to specialist clinicians to select a most appropriate VTE risk assessment score for specific cancer type. Thus the current study was designed and performed to seek the most appropriate one for lung cancer among the authoritative VTE risk assessment scores. Although a single KRS is insufficient to accurately assess the risk of VTE development in patients with lung cancer, a modified KRS can make the difference. Being outstanding in assessment accuracy, clinical applicability and user-friendliness, the ONKOTEV score could be a useful clinical score for clinicians to assess the risk of VTE development among patients with lung cancer in daily clinical practice.

The current study suffers from several limitations. First of all, it was a retrospective study. The prospective validation of the present conclusions among hospitalized and ambulatory lung cancer patients are underway, respectively. Secondly, the time from lung cancer diagnosis to the hospitalization in which data were analyzed among all patients differed from one another, which might introduce the heterogenity of study population. Nevertheless, the median time from lung cancer diagnosis through the hospitalization in which data were analyzed were similar between LC and LC-VTE groups without statistical difference. In addition, since the association between VTE and lung cancer is time-dependent [2], the VTE diagnostic testing approximately one year after lung cancer diagnosis in the present study well reflects the time-dependent VTE prevalence in such patient population. In addition, many of these scores involve the items with respect to cancer therapy. Accordingly, most patients having undergone cancer treatment in the present study suggests that they are suitable candidates of these scores. Thirdly, due to the fragmentary clinical information of ambulatory outpatients with lung cancer in the EMR of participating hospitals, it was intractable to set up a cohort of ambulatory patients as a control group in the present study, albeit it does not affect the results of present study. The last but not least, since the patients being investigated in the current study were all hospitalized medical patients with lung cancer, the results of the current study may not be applicable to ambulatory or surgical patients with lung cancer, or patients with other cancers than lung cancer.

## Conclusions

In conclusion, the current study shows that, among the Khorana score, the PROTECHT score, the CONKO score, the ONKOTEV score, the COMPASS-CAT score, and the CATS/MICA score which are approved by authoritative guidelines, the ONKOTEV score is optimal for the assessment of VTE development in hospitalized medical patients with primary lung cancer. This finding could be conducive to the assessment of VTE development and thromboprophylaxis for hospitalized medical patients with lung cancer. Prospective validation of the present conclusions is warranted in the future.

## Data Availability

The datasets used and/or analysed during the current study are available from the corresponding author on reasonable request.
